# Nutritional management strategies post-liver transplantation: a comparative review of pediatric and adult recipients

**DOI:** 10.3389/fnut.2026.1920355

**Published:** 2026-10-05

**Authors:** Man Wang, Wei Gao, Zhixin Zhang, Meimei Si, Xiangyun Li, Qianyu Wang, Kexin Zhao, Ying Jiang

**Affiliations:** Tianjin First Central Hospital, Tianjin, China

**Keywords:** age stratification, growth and development, nutritional management, post-liver transplantation, sarcopenia

## Abstract

Postoperative malnutrition occurs in 30–60% of liver transplant recipients and impairs perioperative recovery and long-term survival. Distinct malnutrition phenotypes exist across age groups, adult recipients mainly develop sarcopenia driven by sustained hypercatabolism, while pediatric counterparts suffer growth retardation and feeding dysfunction resulting from congenital cholestasis and high anabolic demands. Current studies mostly focus on short-term nutritional support, lacking age-stratified full-cycle intervention covering pre-transplant, early postoperative and long-term maintenance phases, and insufficiently summarize differentiated micronutrient supplementation schemes. This review systematically comparing pathological, metabolic, psychological and nutritional discrepancies between pediatric and adult liver transplant recipients. This review delineates age-specific requirements for macronutrients, fat- and water-soluble vitamins, and trace elements, and summarizes stratified nutritional assessment modalities, enteral and parenteral feeding regimens, and targeted interventions to counteract metabolic adverse effects induced by immunosuppressive agents. Multidisciplinary, staged individualized nutritional regimens are proposed to correct malnutrition, promote catch-up growth in children, preserve muscle mass and reduce metabolic complications in adults, providing evidence-based guidance for clinical precision nutritional management after liver transplantation.

## 1 Introduction

After decades of development, liver transplantation has become the standard treatment for end-stage liver disease. Since the first procedure in 1963, the use of immunosuppressants (1970s–1980s) and living donor and split-liver transplants (1988–1989) have significantly improved success rates (1), and survival rates have continued to rise since the 21st century. Currently, technologies such as mechanical perfusion, artificial intelligence, and multi-omics are driving advancements in the field (2), with the focus shifting toward improving survival rates, expanding indications, and enhancing quality of life (Figure 1). Indications have been extended to include congenital diseases in children and acquired liver diseases in adults, while contraindications have become more stringent (3). The focus has shifted from merely improving survival rates to expanding indications and enhancing long-term quality of life (4).

**Figure 1:**
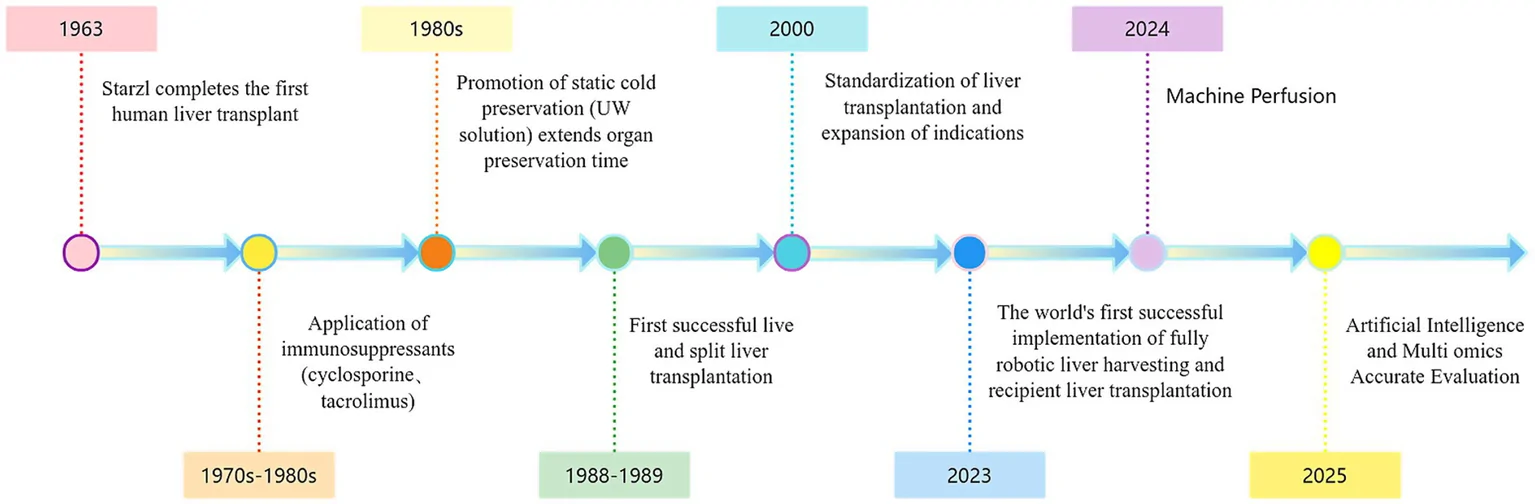
Development history of liver transplantation. This figure is an original illustration by the authors. Since the first human liver transplant in 1963, the field has matured through advancements in organ preservation techniques and immunosuppressive agents, enabling both living donor and split transplants. In the 21st century, standardized surgical protocols and technologies like machine perfusion have further expanded indications. Currently, robotic surgery, artificial intelligence, and multi-omics analysis are propelling liver transplantation into a new era of precision and intelligent management.

Nutrition, as an intervenable prognostic factor, is becoming increasingly recognized for its value. The 5-year survival rate for pediatric liver transplantation is 85–90% (5), while that for adults ranges from 75 to 82% (6). Malnutrition is pervasive throughout all phases of liver transplantation and independently predicts poor prognosis for recipients of all ages. Pre-transplant nutritional impairment is common in pediatric candidates, and 24.4–53.8% still suffer postoperative nutritional disorders characterized by growth retardation related to congenital cholestasis (7, 8). Most adult recipients present preoperative sarcopenia and malabsorption, with postoperative malnutrition incidence ranging from 30.8 to 86.4%, which closely correlates with postoperative infection, complications and long-term mortality (9). Age-related metabolic differences lead to distinct adverse outcomes: pediatric recipients face delayed physical and neurodevelopment due to high anabolic demands and impaired absorption of fat-soluble vitamins, whereas adults develop progressive sarcopenia caused by chronic hypercatabolism and insulin resistance, further elevating infection risks.

This comparative review synthesizes evidence from peer-reviewed original cohort studies, systematic reviews, and international clinical practice guidelines (including ESPGHAN, ESPEN, and AASLD recommendations) published between 2013 and 2025. Literature was identified through PubMed and Web of Science databases using core search terms including liver transplantation, nutritional management, sarcopenia, pediatric growth, and micronutrient supplementation. Findings were stratified by age group (pediatric vs. adult) and clinical stage (pre-transplant, early postoperative, long-term maintenance) to construct a full-cycle comparative framework.

Current studies lack systematic cross-age nutritional management frameworks covering pre-transplant, early postoperative and long-term maintenance stages. Therefore, age-stratified nutritional research is essential to build individualized full-cycle nutritional support systems and improve transplant outcomes. This review compares core differences in nutritional characteristics and intervention strategies between adult and pediatric liver transplant recipients, aiming to provide practical clinical guidance.

## 2 Nutritional requirements differences between children and adults

### 2.1 From a pathological perspective

There are significant differences in the underlying diseases between adult and pediatric liver transplantation, resulting in marked variations in clinical presentation, key clinical considerations, and final outcomes at each stage (5, 9). The primary indications for pediatric liver transplantation are predominantly biliary atresia, as well as hereditary metabolic disorders and congenital hepatic fibrosis (9, 10). These conditions are characterized by congenital, genetic, and cholestatic features, in contrast, the underlying diseases for adult liver transplantation are primarily post-viral hepatitis cirrhosis, alcoholic liver disease, non-alcoholic steatohepatitis, and hepatocellular carcinoma (11, 12), which are predominantly acquired, metabolic, and cirrhotic conditions. These fundamental differences result in distinct nutritional challenges, primarily centered on three core aspects: inadequate intake, malabsorption, and metabolic disorders (9, 13).

In pediatric liver transplant recipients, inadequate intake is primarily caused by feeding difficulties directly resulting from abdominal distension, loss of appetite, portal hypertension, and cholestasis (9), malabsorption, in turn, results from insufficient bile secretion, leading to poor absorption of fats and fat-soluble vitamins, which not only causes severe energy deficiency but also directly impairs skeletal and neurological development (9, 14). Concurrently, persistent systemic inflammation induces a hypermetabolic state, accelerates protein catabolism, and diverts amino acids toward immune responses, further exacerbating energy-protein imbalance and growth arrest (15).

The mechanisms of malnutrition in adult patients are more complex: inadequate intake results from a combination of factors, including zinc deficiency, elevated inflammatory markers, ascites compression, hepatic encephalopathy, and related complications (16), malabsorption is closely associated with drug side effects, surgical impacts, and digestive dysfunction caused by the liver disease itself (13, 16), At the metabolic level, the prominent features are the coexistence of a hypermetabolic state, insulin resistance, and growth hormone resistance, which collectively increase energy expenditure, disrupt glucose and lipid metabolism, and ultimately lead to muscle wasting and malnutrition (15).

In summary, differences in underlying diseases explain the heterogeneity of pre-transplant malnutrition between adults and children: in children, the focus of nutritional care is on growth and development, and malnutrition primarily manifests as growth retardation (9, 14), whereas in adults, the focus is on deterioration of body composition and metabolic imbalance, with the core issues being sarcopenia and severe metabolic disorders (15). These differences are key to implementing precise, effective, and individualized nutritional support strategies before and after transplantation (9, 13).

### 2.2 From the perspective of nutritional composition

Physiological goals and nutritional priorities differ between adult and pediatric liver transplant recipients, and the manifestations and consequences of malnutrition also vary. The core risk of malnutrition in pediatric liver transplant recipients lies in the imbalance between high anabolic demands and insufficient supply, leading to chronic illness-related pediatric malnutrition, with growth faltering and neurodevelopmental impairment as major clinical consequences. Due to cholestasis and high metabolic demands, pediatric liver transplant recipients experience severe deficiencies in energy, protein, and fat-soluble vitamins (A, D, E, and K) (14), which directly lead to specific conditions such as growth retardation, rickets, neurological damage, and coagulation disorders (9). The core intervention strategy involves the use of high-energy, medium-chain triglyceride (MCT)-rich formulas, high-dose supplementation of fat-soluble vitamins, and active measures such as tube feeding to ensure adequate intake, thereby meeting their extremely high nutritional requirements and supporting growth and development (9).

The core risk of malnutrition in adult liver transplant recipients lies in the conflict between high catabolism and nutritional supply, leading to “catabolic malnutrition” characterized by tissue wasting and metabolic disorders (16). Due to high catabolism and malabsorption, adult liver transplant recipients experience core protein and energy deficiencies, manifesting as sarcopenia, accompanied by severe fatigue, weakness, and an increased risk of infection (15). Management should ensure adequate protein intake, emphasize frequent small meals and nighttime snacks, and incorporate resistance training to stimulate muscle synthesis (13), while minimizing the use of glucocorticoids that promote muscle breakdown as much as possible (17).

Clinical practice guidelines issued by ESPGHAN and ESPEN advocate for MCT-enriched enteral formulas to optimize energy delivery in children with biliary atresia. MCTs are absorbed independently of bile, providing rapid energy, while also supporting the supplementation of water-soluble and fat-soluble vitamins. The reference daily energy requirement for infants with biliary atresia in the pre-transplant period is 130–150 kcal/kg, which is 30–50% higher than that of age-matched healthy infants. This value decreases with advancing age, and nutritional requirements must be individualized based on nutritional status, growth velocity, graft function, clinical condition, and measured energy expenditure when available (18). Energy intake for pediatric liver transplant recipients must reach at least 120% of basal energy expenditure, which can be achieved through oral or nasogastric administration of high-energy enteral formulas (9). Supplementation with essential fatty acids such as omega-3 and omega-6 is necessary, as these can modulate inflammation and immune function while supporting brain and visual development (14).

Differences in nutritional requirements between adult and pediatric liver transplant recipients (Table 1) directly result in fundamental distinctions in the spectrum of malnutrition risks and clinical manifestations, clinical nutritional support strategies must be highly targeted (9): Children require aggressive, high-intake supplementation of nutrients such as medium-chain triglycerides, omega-3, and omega-6 to maintain growth trajectories (14), adults, in contrast, require a strategy that ensures precise and adequate protein intake while addressing complications, to suppress catabolism and promote tissue repair (13, 15). Recognizing these differences is fundamental to the prevention and treatment of liver transplant-related malnutrition and the improvement of long-term outcomes.

**Table 1:** Reference strategies for nutritional supplementation in liver transplant recipients.

Nutritional requirements		Various types of liver diseases in adults				Children’s liver transplantation
		Liver cirrhosis	Alcoholic liver disease	Hepatocellular carcinoma	Non alcoholic fatty liver disease	
Macronutrients	Energy	Compensatory phase: 25–35 kcal/kg/day Decompensation period: 35-40 kcal/kg/day	35-45 kcal/kg/day	25–35 kcal/kg/day	Create negative balance and lose 3–10% weight	130-150 kcal/kg
	Carbohydrate	Provide 50–60% of the total energy		Provide 45–60% of the total energy	Strictly limit the addition of sugar and fructose	6-8 g/kg/day
	Protein	1.2–1.5 g/kg/day		1.2–2.0 g/kg/day	Adequate intake of high-quality protein	2.5-3 g/kg/day
	Fat	Provide 25–35% of the total energy		Provide 25–40% of the total energy	Replace saturated fat with unsaturated fat	5-6 g/kg/day
Micronutrients	Vitamin D	800-2000 IU				10-50 ng/kg/day
	Calcium	1,000-1500 mg				50-100 mg/kg/day
	Zinc	15-30 mg				1 mg/kg/day
	Magnesium	200-400 mg				5-10 mg/kg/day
	Iron	Only consider supplementation when there is iron deficiency anemia, stable transplanted liver function, and no active infection				3-6 mg/kg/day

### 2.3 From a metabolic perspective

In children, metabolic characteristics include fat malabsorption, severe energy deficiency, and deficiencies in fat-soluble vitamins, whereas in adults, metabolic characteristics manifest as complex malabsorption, simultaneous impairment of multiple nutrient absorptions, and protein-energy malnutrition, often accompanied by trace element deficiencies (9, 13).

Children exhibit characteristics of high anabolism and growth-driven metabolism, primarily manifested as bile stasis-related impairments in fat and fat-soluble vitamin absorption, which directly affect growth and development. Children with chronic liver disease have a basal metabolic rate 30–40% higher than that of healthy children. Their metabolic profile is characterized by extremely high anabolic demands for growth and development, compounded by energy expenditure associated with liver disease. The core objective of nutritional support is to provide above-normal levels of energy and macronutrients to meet the triple demands of basal metabolism, stress recovery, and linear growth and organ development. Therefore, pediatric nutritional formulas should be adjusted to medium-chain triglyceride (MCT) formulations, in which MCTs may account for up to 80% of total fat (9). High-energy-density formulas (1–1.5 kcal/mL) should be used, with routine supplementation of water-soluble and fat-soluble vitamins, fortified calcium and phosphorus supplementation, and appropriate supplementation of essential fatty acids (14).

Adult liver transplant recipients, due to long-term catabolism resulting from cirrhosis, exhibit a state of hypercatabolism characterized by massive protein breakdown, insulin resistance, and loss of lean body mass, the transplant procedure further exacerbates this process (16). The core of nutritional impairment stems from complex malabsorption triggered by factors such as portal hypertension (13): cholestasis leads to bile salt deficiency, impairing the absorption of fats and fat-soluble vitamins, portal hypertension induces intestinal mucosal lesions and bacterial overgrowth, some patients also suffer from exocrine pancreatic insufficiency. Nutritional support should focus on counteracting catabolism and promoting repair, providing adequate energy, using semi-elemental formulas containing peptides and medium-chain triglycerides, and emphasizing monitoring of body weight, plasma proteins, and electrolytes (15).

### 2.4 From a psychological perspective

The impact of psychological factors on the nutritional status of liver transplant recipients also varies by age, with these differences primarily stemming from differences in cognitive maturity, psychological coping strategies, and dependency between the two groups (13, 19).

Adult liver transplant recipients possess full cognitive capacity, and the mechanisms underlying their malnutrition primarily manifest as cognitively driven implicit eating disorders (19). Disease-related dietary restrictions (such as strict control of protein or potassium and sodium intake) can easily induce intense psychological stress, which interacts with anxiety and depression to form two primary pathways of influence: first, the restriction pathway, where overinterpretation of medical advice or anxious compliance may evolve into anorexia-like restrictive eating behaviors, leading to severe calorie and protein deficiencies that exacerbate sarcopenia and frailty (16), second, the emotional pathway, where negative emotions may trigger emotional eating or binge-eating behaviors, leading to dependence on or a tendency to abuse specific foods. Therefore, adult malnutrition arises primarily from abnormal eating behaviors induced by personal cognitive and emotional disturbances (19).

The mechanisms of malnutrition in pediatric liver transplant recipients are more dependent and exogenous in nature (14). Due to immature cognitive development, children’s nutritional status is primarily influenced by the psychological state and cognitive level of their caregivers (9). The severe illness of the child and the stress, anxiety, and burnout experienced by caregivers during the transplant process can impair their ability to implement complex nutritional support regimens, leading to inadequate dietary management within the home environment, Furthermore, caregivers’ limited knowledge of medical nutrition and insufficient professional support often result in inappropriate feeding practices, indirectly contributing to pediatric malnutrition (9, 14). Therefore, malnutrition in children is primarily shaped by the combined psychological, cognitive, and behavioral factors of the caregivers, rather than by the children themselves.

In summary, malnutrition in adult liver transplant recipients primarily stems from psychologically driven abnormal eating behaviors, whereas in children, it is more heavily influenced by the indirect effects of caregivers’ psychological and cognitive burdens (9, 16). This fundamental difference necessitates targeted clinical intervention strategies: adults require enhanced psychological screening and cognitive-behavioral interventions (19), while pediatric cases must prioritize family support and caregiver education as the core of nutritional management (9, 14).

## 3 Micronutrient requirements for pediatric and adult liver transplant recipients

Micronutrient deficiencies (fat-soluble vitamins, water-soluble vitamins, trace elements) are universal across all stages of liver transplantation, with distinct demand characteristics between pediatric and adult liver transplant recipients (Table 1).

### 3.1 Fat-soluble vitamins (A/D/E/K)

Pediatric recipients with cholestasis suffer severe bile salt insufficiency, leading to impaired absorption of fat-soluble vitamins, as micelle formation required for lipid-soluble nutrient uptake is severely restricted in cholestatic conditions (20). Routine high-dose supplementation is mandatory: vitamin D 800–1,200 IU/d, vitamin A 5000–10,000 IU/d, vitamin E 15–25 IU/kg/d, vitamin K 0.3–0.5 mg/kg/d (14). Deficiencies cause rickets, neurological degeneration and coagulation dysfunction, which directly interfere with childhood growth and neurodevelopment, nearly 70% of cholestatic pediatric candidates awaiting liver transplant present concurrent vitamin A and D depletion.

Adult recipients develop mild to moderate fat-soluble vitamin malabsorption due to chronic cirrhosis and intestinal mucosal lesions induced by portal hypertension and chronic inflammation (21). Standard supplementation doses are lower than children: vitamin D 600–800 IU/d, vitamin K 100–200 μg/d, with regular serum vitamin concentration monitoring to avoid hepatotoxicity caused by excessive vitamin A accumulation, as hepatic storage capacity is compromised in cirrhotic patients. The ESPEN clinical nutrition guideline for chronic liver disease recommends quarterly serum fat-soluble vitamin screening for all adult transplant recipients long-term post-liver transplantation.

### 3.2 Water-soluble vitamins

B vitamins participate in energy metabolism and immunosuppressant drug metabolism, facilitating hepatic clearance of calcineurin inhibitors and mTOR inhibitors (22). All recipients require routine full-spectrum B-complex supplementation. Pediatric recipients have higher daily requirements of B12 and folate to support cell proliferation and organ development during rapid growth phases (14), adult recipients with sarcopenia prioritize vitamin B6 and niacin to improve muscle energy metabolism and attenuate protein catabolism induced by glucocorticoids (21). Vitamin C supplementation (100–200 mg/d) is recommended for all recipients to reduce oxidative stress and surgical inflammatory response, especially within the first 3 months post-transplantation (23).

### 3.3 Essential trace elements

*Zinc:* Zinc deficiency suppresses appetite, immune function and protein synthesis, with prevalence exceeding 85% in decompensated cirrhotic candidates pre-transplant (24). Pediatric demand: 1–2 mg/kg/d; adult standard dose: 15–25 mg/d. Zinc supplementation is essential for recipients with ascites and poor oral intake, as diuretic therapy accelerates urinary zinc loss in adult patients (25).

*Selenium:* Selenium regulates antioxidant capacity, reducing infection risk after transplantation, with uniform daily demand of 50–100 μg for both age groups. Low serum selenium correlates with elevated post-transplant sepsis incidence and worsened oxidative liver injury in longitudinal cohort studies (26).

*Calcium and phosphorus:* Core for bone health. Pediatric recipients need calcium 800–1,500 mg/d plus phosphorus matching growth velocity to avoid transplant-related osteopenia; adults require 800–1,000 mg calcium daily to counteract glucocorticoid-induced osteoporosis, a complication documented in over 60% of long-term adult liver transplant survivors (27).

*Iron:* Iron supplementation is restricted to recipients with confirmed iron-deficiency anemia; excessive iron overload aggravates liver oxidative injury, especially for adult recipients with non-alcoholic fatty liver disease, and elevates post-transplant infectious complication risk (28).

### 3.4 Clinical management principles of micronutrients

Micronutrient supplementation adopts individualized dynamic adjustment: pediatric recipients receive monthly serum micronutrient testing during pre-transplant and early postoperative stages, while adults conduct quarterly screening consistent with NASPGHAN and ESPEN consensus recommendations (27). MCT fat formula is matched with fat-soluble vitamin supplements to improve absorption efficiency, bypassing impaired bile salt micellization in cholestatic recipients (20). Long-term immunosuppressant users require lifelong low-dose micronutrient maintenance to prevent persistent deficiency-induced complications including bone loss, muscle wasting and impaired growth in children.

## 4 Optimal nutritional supplementation management for pediatric and adult liver transplant recipients

From infants to adults, the comprehensive management of malnutrition following liver transplantation requires multidimensional nutritional support and assessment, physical exercise, and mental health guidance to reduce potential damage associated with nutritional deficiencies (Figure 2) (13). Significant differences exist between adult and pediatric populations, primarily due to the metabolic shift from a highly anabolic to a highly catabolic state. Ideally, this management should be supported by a multidisciplinary team comprising dietitians, exercise specialists, and others, while also considering the impact of medications on metabolism, particularly commonly used immunosuppressive therapies (17).

**Figure 2:**
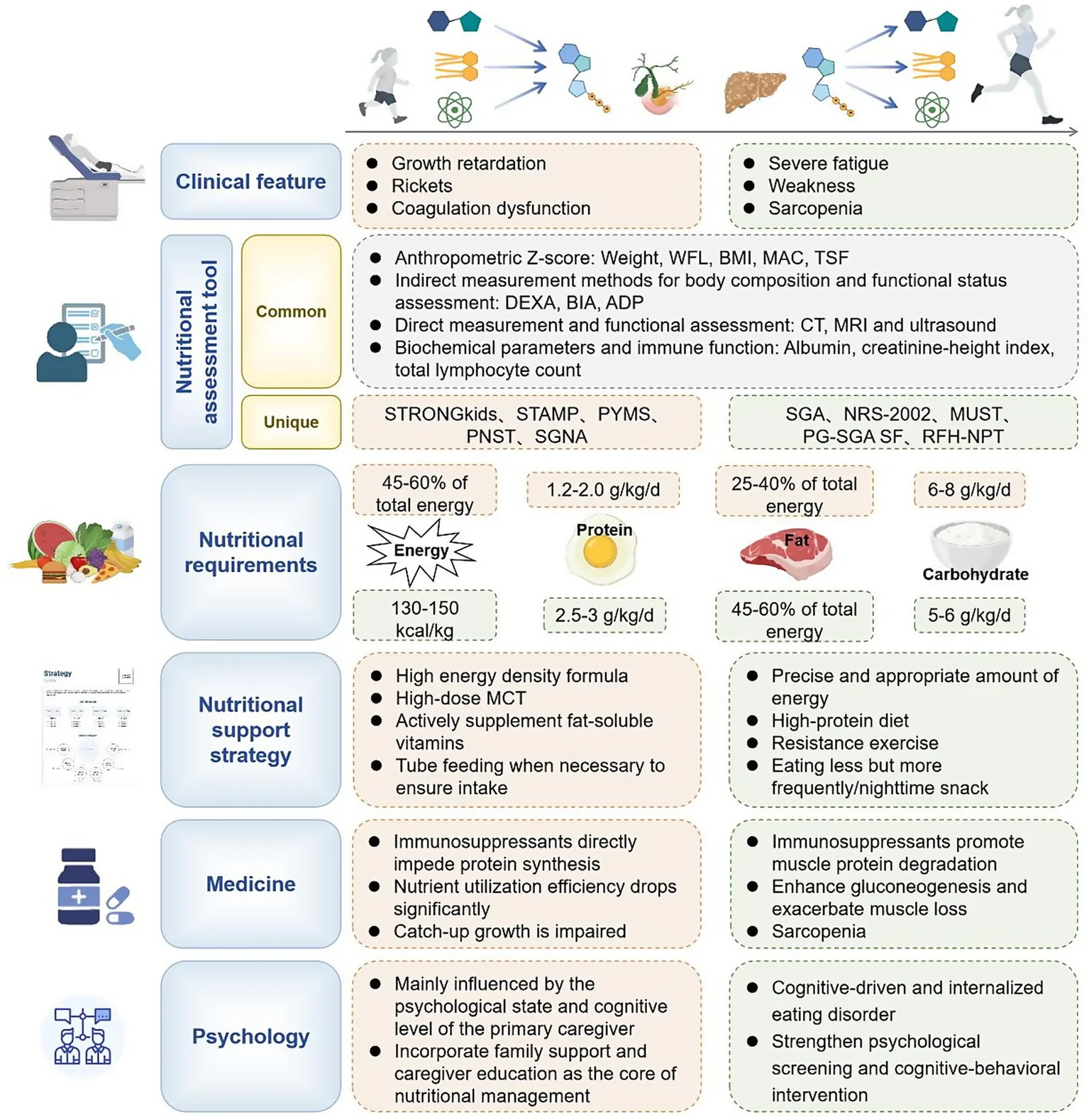
Nutritional differences between adult and child liver transplantation. This figure is an original illustration by the authors. Nutritional management for pediatric liver transplant recipients focuses on catch-up growth, requiring high-energy and high-protein support alongside family-based feeding guidance. Adult patients primarily experience sarcopenia and fatigue, necessitating precise energy intake combined with resistance training to counteract catabolism. Both groups exhibit age-specific differences in assessment tools, nutritional requirements, and psychosocial interventions, reflecting tailored strategies addressing distinct physiological and metabolic characteristics.

### 4.1 Assessment

Nutritional assessment of liver transplant candidates is central to prognostic prediction and intervention, since the ultimate goal of assessment is to optimize pre- and post-transplant outcomes, various assessment criteria share common elements (9, 13). Both adult and pediatric liver transplantation commonly utilize anthropometric Z-scores, indirect measurements of body composition and functional status, adjunctive direct imaging assessments (computed tomography [CT], magnetic resonance imaging [MRI], ultrasound), deuterium dilution, whole-body potassium measurement, and biochemical markers (13, 15). Of note, CT and MRI have limited validation in pediatric liver transplant populations, with no universally accepted reference ranges or diagnostic cutoffs for this specific group. CT involves ionizing radiation exposure that requires risk–benefit justification in children, while MRI is limited by cost, availability, long scan duration, and potential need for sedation in young children. Thus, they are not recommended as routine nutritional screening tools but may be used for adjunctive body composition evaluation when clinically indicated. However, because children are in a dynamic phase of growth and development, while adults are in a relatively stable phase of metabolic maintenance, each group has its own specific assessment tools (9).

Nutritional screening tools for pediatric liver transplantation typically focus on growth and development indicators and assessments of family feeding practices. Commonly used initial screening tools include STRONGkids and the Pediatric Malnutrition Assessment Screening Tool (STAMP). It should be noted that these tools have been validated primarily in heterogeneous hospitalized pediatric populations, not specifically in pediatric liver transplant candidates or recipients, and lack transplant-specific diagnostic validation. They serve as useful first-line screening aids but cannot replace comprehensive nutritional assessment performed by clinical nutrition specialists (9, 14). In contrast, screening tools for adult liver transplantation place greater emphasis on disease-related metabolic demands, surgical risk assessment, and complication prediction. Core tools specific to adult liver transplantation include the Subjective Global Assessment (SGA), Nutrition Risk Screening 2002 (NRS-2002) (13), all of which are standardized tools recommended by authoritative journals such as Clinical Nutrition and Hepatology (15, 29) (Table 2).

**Table 2:** Differences in nutritional assessment and supplementation between adult and child liver transplantation.

Category		Adult	Minor
Evaluation tool	Shared	1. Anthropometry Z-score: weight, length to weight ratio (WFL), BMI, upper arm circumference (MAC), and triceps skinfold thickness (TSF). 2. Indirect measurement methods for assessing body composition and functional status: Dual energy X-ray absorptiometry (DEXA), bioelectrical impedance analysis (BIA), air displacement plethysmography (ADP). 3. Direct measurement and functional evaluation (CT, MRI, and ultrasound), deuterium dilution method, whole body potassium count. 4. Biochemical parameters and immune function: serum transferrin and retinol binding protein, albumin (half-life 17–21 days), prealbumin (half-life 2–3 days, more sensitive), creatinine body high index, total lymphocyte count.	
	Unique	SGA, NRS-2002, MUST, PG-SGA SF, RFH-NPT	STRONGkids, STAMP, PYMS, PNST, SGNA
Nutritional supplementation	Nutrition target	1. Maintain nitrogen balance and prevent muscle breakdown. 2. Support liver regeneration and reduce the risk of postoperative infection. 3. Control blood sugar and blood lipids.	1. Correcting weight, height, and physical developmental delays (z score improvement). 2. Promote bone metabolism and endocrine balance. 3. Prevent long-term obesity or muscle wasting (postoperative body composition monitoring).
	The timing for initiating nutritional interventions	Start enteral/intravenous nutrition immediately after surgery to prevent catabolism caused by postoperative high metabolic status.	Before surgery, it is necessary to actively correct malnutrition, especially weight and height deficiencies caused by chronic liver disease; Early enteral feeding after surgery is also common, but often accompanied by long-term growth and development goals.
	Main nutritional formula	1. Intravenous nutrition often includes branched chain amino acid (BCAA) formula. 2. Supplementation with omega-3 fish oil (omega-3) has been proven to improve liver function and inflammation. 3. Compared with a simple glucose solution, the BCAA+ω 3 regimen is more beneficial.	1. A formula high in calories and rich in medium chain triglycerides (MCT) to increase energy density and improve fat absorption. 2. Essential fat soluble vitamins (A, D, E, K) and essential trace element supplements. 3. For children who cannot eat through oral/nasogastric tubes, use total parenteral nutrition (PN) and add omega-3 fat emulsion or bile acid salts if necessary.
Nutritional supplementation	Support strategy	1. Extraintestinal nutrition: Adult liver transplant PN is more commonly used as a supplement when EN is contraindicated or insufficient, emphasizing the avoidance of overfeeding and prevention of catheter-related infections. 2. Feeding mode: For adult liver transplantation, it is strongly recommended to eat small meals frequently and have additional meals at night.	1. Enteral nutrition: introducing complementary foods from 6 months of liver transplantation in children. Extraintestinal nutrition: mainly includes 24-h continuous infusion, strict fluid management, and electrolyte adjustment. 2. Feeding mode: Children’s liver transplantation focuses more on ensuring total intake through tube feeding or high-energy density formula.
	Common complications and countermeasures	1. The risk of venous nutrition related liver disease (PNALD) is relatively low. 2. Avoid excessive calorie intake that can lead to high blood sugar.	1. The incidence of PN related liver disease (PNALD) is relatively high, and it is necessary to control the total calorie and fat/glucose ratio. 2. Essential fatty acids and bile acid salts must be supplemented to prevent bile stasis.
	Long term follow-up focus	Pay attention to metabolic syndrome and cardiovascular risk	Pay attention to growth curves, bone density, and changes in body composition (obesity or sarcopenia)

The goals of nutritional support in pediatric liver transplantation are to promote growth and development, correct malnutrition related to absorption, meet the demands of normal growth, neurocognitive development, and organ maturation, and achieve catch-up growth comparable to that of healthy children (9, 14), whereas the goals of nutritional support in adult liver transplantation are to maintain body composition and function, with a core focus on correcting and preventing sarcopenia, preserving lean body mass, improving functional status, and reducing the risk of complications before and after transplantation (13, 15). Consequently, the pathways and strategies for nutritional support in adult and pediatric liver transplantation have distinct priorities, primarily reflected in differences in enteral nutrition, parenteral nutrition, and feeding methods. Furthermore, both enteral and parenteral nutrition for liver transplant recipients must be individually tailored according to age-specific characteristics (9).

Early enteral nutrition is a common core component of postoperative management for both adults and children, aimed at maintaining intestinal barrier integrity and reducing the risk of bacterial translocation and infection (13). Pediatric patients often experience malabsorption and growth retardation due to congenital biliary tract diseases. Post-liver transplantation, high-energy-density formulas must be initiated promptly, and frequent feeding via nasogastric or gastrostomy tubes is required to ensure adequate energy and protein intake. Concurrently, emphasis is placed on cultivating oral sensory and motor functions through the timely introduction of complementary foods (9, 14). In pediatric liver transplant recipients, parenteral nutrition serves primarily as a critical means of sustaining life and promoting growth, particularly during the end-stage liver disease phase prior to transplantation, where continuous infusion and strict management of fluid and electrolyte balance are required (9). In adult patients, however, parenteral nutrition is mainly used as a supplement when enteral nutrition is insufficient or contraindicated, and vigilance is required regarding overfeeding and catheter-related complications (13, 16).

Regarding feeding strategies, adult patients benefit from frequent small meals and nighttime supplements to minimize fasting periods and inhibit muscle breakdown (15), for children, the emphasis is on ensuring nutritional requirements per kilogram of body weight through scheduled tube feeding or high-energy formulas to support catch-up growth (9, 14). In summary, nutritional support for pediatric liver transplantation centers on high-energy, high-protein, and micronutrient management (20), whereas adult nutritional support focuses on combating sarcopenia, maintaining physical function through a combination of nutrition and exercise (13, 15) (Table 2).

### 4.2 Immunotherapy

While immunosuppressive therapy ensures graft function following liver transplantation, its metabolic side effects can lead to long-term malnutrition in patients (17). Because children and adults have significant differences in physiological and metabolic needs, the impact of immunosuppressants on nutritional status and clinical manifestations varies among these populations (9, 17).

The primary nutritional goal for pediatric liver transplant recipients is to support growth and development, a process that is highly dependent on sustained protein synthesis (14). The mammalian target of rapamycin (mTOR) signaling pathway is a key regulatory hub for cell growth and proliferation, mTOR inhibitors such as sirolimus directly inhibit this pathway, thereby impeding protein synthesis and tissue formation (17). Even with adequate nutritional support (protein intake of 3–4 g/kg/day), the anabolic suppression induced by these drugs significantly reduces nutrient utilization efficiency, clinically manifesting as impaired catch-up growth (9, 17). Long-term follow-up data show that more than 20% of pediatric patients experience growth and developmental disorders, which is closely related to the suppression of growth potential by immunosuppressants during the critical growth period of 2–3 years after transplantation (14, 17).

Nutritional challenges in adult liver transplant recipients primarily manifest as muscle wasting under a state of high catabolism, immunosuppressants such as glucocorticoids activate the ubiquitin-proteasome system, promoting muscle protein degradation and enhancing gluconeogenesis, which further exacerbates muscle loss (15, 17). Although the recommended protein intake is 1.5–2.0 g/kg/day, adult liver transplant recipients often struggle to achieve a positive nitrogen balance, leading to sarcopenia, which clinically manifests as fatigue, weakness, and an increased risk of infection (15, 16).

Given these differences, nutritional management strategies must be highly individualized (9): pediatric liver transplant recipients require enhanced nutritional support to overcome anabolic suppression through high-energy, high-protein intake (9, 14), and immunotherapy regimens should prioritize medications with minimal impact on growth, with close monitoring of growth curves (17), In contrast, adult liver transplant recipients require a combination of adequate protein intake and resistance training to stimulate muscle (14). Whenever clinically feasible, glucocorticoids should be minimized or discontinued, and nutritional outcomes should be assessed primarily through functional indicators such as grip strength and muscle mass (13–15).

### 4.3 Stage-based nutritional management: pre-transplant, early post-transplant, long-term maintenance

Nutritional intervention for pediatric and adult liver transplant recipients exhibits distinct goals and operational strategies across three critical clinical stages: pre-transplant waiting period, early postoperative phase (within 3 months after transplantation), and long-term stable maintenance (≥1 year post-transplant).

#### 4.3.1 Pre-transplant nutritional management

The pre-transplant stage is the core window to reverse baseline malnutrition and optimize surgical tolerance. For pediatric liver transplant recipients, most carry congenital cholestatic disease. Clinicians prioritize high-energy MCT enteral nutrition to reverse growth retardation (30), routinely supplement fat-soluble vitamins, and correct feeding difficulties via gastrostomy tube placement for children with severe anorexia. Target energy intake is maintained at 130–150 kcal/kg/day to achieve catch-up growth before surgery (31). For adult liver transplant recipients, sarcopenia and insulin resistance are dominant impairments. Pre-transplant intervention targets nitrogen balance recovery: protein intake is adjusted to 1.2–1.5 g/kg/d (31), combined with low-resistance aerobic training to preserve lean muscle mass. Restrictive sodium and lipid intake is customized for patients with ascites, hepatic steatosis and hepatocellular carcinoma, while trace element deficiencies (zinc, selenium) are corrected to reduce surgical infection risk.

#### 4.3.2 Early post-transplant nutritional management (0–3 months)

This stage is characterized by surgical stress, immunosuppressant initiation and intestinal function recovery, with the core goal of restoring intestinal barrier function. Early enteral nutrition is recommended for hemodynamically stable recipients with intact gastrointestinal function. For patients undergoing duct-to-duct biliary reconstruction, initiation is typically feasible within 24–48 h after liver transplantation (30). For recipients receiving Roux-en-Y hepaticojejunostomy, initiation of enteral feeding may be delayed depending on surgical recovery, gastrointestinal function, and hemodynamic stability, consistent with clinical practice guidelines that note differing timelines based on biliary reconstruction technique (32). Pediatric recipients adopt continuous nasogastric tube feeding with high-density formula to compensate perioperative energy loss, while gradual oral feeding training is launched to rebuild feeding coordination (33). Adult recipients adopt frequent small meals plus bedtime protein supplements to restrain postoperative hypercatabolism and reduce muscle breakdown. Parenteral nutrition is only reserved for recipients with severe intestinal paralysis, anastomotic complications or feeding intolerance, with strict monitoring to avoid overfeeding-induced liver burden (31).

#### 4.3.3 Long-term post-transplant nutritional management (≥1 year)

Long-term management focuses on offsetting the metabolic side effects of lifelong immunosuppressants and preventing chronic metabolic complications. Pediatric recipients require lifelong dynamic growth monitoring; growth curves are tracked quarterly, with timely elevation of energy and protein supplementation if growth velocity declines due to mTOR inhibitor therapy (7). Regular calcium, vitamin D and phosphorus supplementation prevents long-term osteopenia. Adult recipients face high risks of new-onset metabolic syndrome, obesity and progressive sarcopenia. Long-term protocols combine standardized protein intake with weekly resistance training, while lipid and carbohydrate intake are controlled to counteract glucocorticoid-induced hyperglycemia and lipid disorder (34). Psychological nutritional intervention is regularly conducted to avoid restrictive eating or emotional overeating (35).

## 5 Conclusion

This comparative review systematically delineates distinct pathological, metabolic, psychological, and nutritional phenotypes between pediatric and adult liver transplant recipients across the full perioperative continuum. Pediatric recipients face predominant risks of growth faltering and fat-soluble vitamin deficiency driven by congenital cholestasis and high anabolic demands, while adult recipients primarily develop sarcopenia and metabolic disorders secondary to chronic hypercatabolism and insulin resistance. These fundamental differences necessitate age-stratified strategies for nutritional assessment, micronutrient supplementation, enteral/parenteral feeding regimens, and management of immunosuppressant-related metabolic adverse effects.

Implementation of staged, individualized nutritional management-with a focus on catch-up growth and caregiver support in children, and sarcopenia prevention and metabolic control in adults-can improve postoperative recovery and long-term transplant outcomes. However, current evidence has notable limitations: several commonly used nutritional assessment tools lack transplant-specific validation in pediatric populations, evidence for age-specific dosing of micronutrients remains heterogeneous, and long-term follow-up data linking early nutritional programming to adult metabolic health are scarce. Further high-quality, age-stratified clinical studies are needed to refine precision nutritional protocols for liver transplant recipients.

### 5.1 Future directions

Future research on nutrition for liver transplantation should focus on personalized, precision-based support, refining patient subtypes based on etiology, metabolism, and microbiome characteristics, and developing dynamic treatment plans. The long-term effects of early nutritional programming warrant further investigation, follow-up studies spanning childhood through adulthood are needed to clarify its value in preventing metabolic syndrome. New formulations are urgently needed: children require lipid emulsions with high absorption efficiency and low hepatotoxicity (36), while adults should explore specific nutrients that promote muscle synthesis and synergistic models with resistance training (37). Psychological and behavioral interventions must be integrated with technological support to establish psychological screening pathways for adults and remote support systems for caregivers of children. Additionally, the management of interactions between immunosuppressants and nutrition should be optimized to mitigate drug-induced muscle wasting or growth suppression. In the future, precise, comprehensive, and multidisciplinary strategies are needed to improve the long-term quality of life for patients of all ages.
